# *CUL4A*, *ERCC5*, and *ERCC1* as Predictive Factors for Trabectedin Efficacy in Advanced Soft Tissue Sarcomas (STS): A Spanish Group for Sarcoma Research (GEIS) Study

**DOI:** 10.3390/cancers12051128

**Published:** 2020-04-30

**Authors:** David S. Moura, Paloma Sanchez-Bustos, Antonio Fernandez-Serra, María Lopez-Alvarez, José L. Mondaza-Hernandez, Elena Blanco-Alcaina, Angela Gavilan-Naranjo, Paula Martinez-Delgado, Serena Lacerenza, Paloma Santos-Fernandez, Irene Carrasco-Garcia, Samuel Hidalgo-Rios, Antonio Gutierrez, Rafael Ramos, Nadia Hindi, Miguel Taron, Jose Antonio Lopez-Guerrero, Javier Martin-Broto

**Affiliations:** 1 Institute of Biomedicine of Sevilla (IBIS, HUVR, CSIC, Universidad de Sevilla), 41013 Sevilla, Spain; david.moura@usal.es (D.S.M.); sanchezbustospaloma@gmail.com (P.S.-B.); marlopalv4@gmail.com (M.L.-A.); joseluciniomondaza@gmail.com (J.L.M.-H.); elena.blancoalcaina@gmail.com (E.B.-A.); amgavnar@gmail.com (A.G.-N.); paula.mrtnez.delgado@gmail.com (P.M.-D.); serelac@hotmail.it (S.L.); paloma.santos.fdez@gmail.com (P.S.-F.); irenecg1990@gmail.com (I.C.-G.); hidalgosamuel@hotmail.com (S.H.-R.); nhindi@mustbesevilla.org (N.H.); taron.miquel@gmail.com (M.T.); 2 Laboratory of Molecular Biology, Fundación Instituto Valenciano de Oncología, 46009 Valencia, Spain; afernandez@fivo.org (A.F.-S.); jalopez@fivo.org (J.A.L.-G.); 3 Medical Oncology Department, University Hospital Virgen del Rocio, 41013 Sevilla, Spain; 4 Hematology Department, University Hospital Son Espases/IdISBa, 07120 Mallorca, Spain; antoniom.gutierrez@ssib.es; 5 Pathology Department, University Hospital Son Espases, 07120 Mallorca, Spain; rafaelf.ramos@ssib.es; 6 Synlab Diagnosticos Globales SAU, 28108 Madrid, Spain; 7 Department of Basic Medical Sciences, School of Medicine, Catholic University of Valencia ‘San Vicente Martir’, 46001 Valencia, Spain

**Keywords:** trabectedin, ERCC1, CUL4A, predictive biomarkers, soft-tissue sarcoma

## Abstract

A translational study was designed to analyze the expression of nucleotide excision repair (NER) and homologous recombination (HR) genes as potential predictive biomarkers for trabectedin in soft-tissue sarcoma (STS). This study is part of a randomized phase II trial comparing trabectedin plus doxorubicin versus doxorubicin in advanced STS. Gene expression levels were evaluated by qRT-PCR, while CUL4A protein levels were quantified by immunohistochemistry. Expression levels were correlated with patients’ progression-free survival (PFS) and overall survival (OS). Gene expression was also evaluated in cell lines and correlated with trabectedin sensitivity. In doxorubicin arm and in the whole series, which includes samples from both arms, no significant differences in terms of PFS were observed amongst the analyzed genes. In the group treated with trabectedin plus doxorubicin, the median of PFS was significantly longer in cases with *CUL4A*, *ERCC1,* or *ERCC5* overexpression, while *BRCA1* expression did not correlated with PFS. Gene expression had no prognostic influence in OS. CUL4A protein levels correlated with worse PFS in doxorubicin arm and in the whole series. In cell lines, only overexpression of *ERCC1* was significantly correlated with trabectedin sensitivity. In conclusion, *CUL4A, ERCC5,* and mainly *ERCC1* acted as predictive factors for trabectedin efficacy in advanced STS.

## 1. Introduction

Trabectedin is a tetrahydroisoquinoline alkaloid approved in 2007, for the treatment of adult patients with advanced soft-tissue sarcoma (STS), after failure of anthracyclines and ifosfamide, or patients unsuited to receive these agents. Trabectedin has a wide spectrum of mechanisms of action in the tumor and in the microenvironment. As a DNA-binding agent, it interferes with gene transcription and with the DNA repair machinery, leading to DNA damage accumulation and cell cycle perturbation, with a delay in S phase progression and accumulation of cells in G2 phase [1,2,3]. More importantly, trabectedin interacts with the nucleotide excision repair (NER) machinery to exert its antitumor activity [4]. Yet and although the mechanisms of action of trabectedin have been comprehensively described, there are only few potential predictive biomarkers of drug activity [5,6].

Few retrospective studies have shown that NER- and homologous recombination (HR)-associated genes could be potential predictive factors for trabectedin efficacy in STS [5,7,8,9]. Trabectedin seems to bind to specific triplets of the DNA minor groove, projecting out of the DNA a part of its structure, which probably traps other proteins at the site of adduct such as XPG (*ERCC5*), forming large ternary complexes. Of note, trabectedin seems to be more active in the context of high levels of expression of NER genes (*ERCC1* and *ERCC5*) and low expression levels of HR genes (*BRCA1*) [8,9,10]. NER-deficient cells are generally more resistant to trabectedin treatment [11]. Noteworthy, trabectedin produces DNA double-strand breaks, which associated with the impaired damage repair present in some sarcomas, through HR deficiency or BRCAness-like phenotype, leads to the rapid cell death of cancer cells; in a concept known as synthetic lethality [12].

Moreover, DNA-damage binding proteins (i.e., DDB1 and DDB2), which are known components of NER pathway [13] have been described to be part of the CUL4A ubiquitin ligase complex, suggesting a link between NER and the ubiquitin–proteasome pathway (UPP). CUL4-DDB1 E3-ligase complex seems to regulate the proteolysis of key proteins responsible for DDR [13]. In fact, after DNA damage, DDBs proteins form a complex that targets histones towards their ubiquitination and degradation during NER. This degradation induces chromatin remodeling and the activation of NER pathway, by recruiting XPC-containing complex that recognizes DNA lesions [14,15]. Therefore, the expression of CUL4A could be an indicator of NER pathway integrity and trabectedin efficacy. In line with this, high expression of CUL4A has been associated with trabectedin activity, suggesting that this gene/protein could be a predictive biomarker of drug efficacy [7,16].

The aim of this study was to analyze the expression of NER and HR genes (i.e., *ERCC1*, *ERCC5,* and *BRCA1*), as well as *CUL4A*, as potential predictive factors of trabectedin efficacy in STS. These genes were selected from bibliography, where they were described as potential predictive biomarkers for trabectedin [7,8,16,17,18]. This prospective translational analyses were performed as a correlative study within the comparative phase II trial that compared trabectedin plus doxorubicin versus doxorubicin alone as first line of advanced STS [19].

## 2. Results

### 2.1. Demographic and Pathologic Features

A cohort of 66 cases, derived from the randomized phase II study of trabectedin and doxorubicin compared with doxorubicin alone as first-line treatment in patients with advanced STS, were included in the translational study associated with the clinical trial. All the patients included in the translational study had FFPE tumor samples available, with enough tissue (i.e., derived from surgery) for gene expression analysis. Of those initially included in the clinical trial (*n* = 113), 47 were removed from the translational study due to the lack of enough FFPE tumor tissue. All the patients were enrolled in the trial between November 2009 and October 2012, at the time of clinical cut-off the median of follow-up was 13 months.

The median age of the subset of patients included in the translational research was 52 years (21–72); 31 (47%) being females and 35 (53%) males. Median tumor size was of 90 mm (2–300 mm). Primary tumor sites were: Extremities (38.4%), head and neck (3.1%), truck wall (4.6%), retroperitoneum (23.1%), and other sites (30.8%). This translational study includes several subtypes: Leiomyosarcoma (*n* = 22), liposarcoma (*n* = 12), undifferentiated pleomorphic sarcoma (UPS; *n* = 12), fibrosarcoma (*n* = 4), hemangiopericytoma (*n* = 3), malignant peripheral nerve sheath tumor (MPNST; *n* = 3), synovial sarcoma (*n* = 3), and others (*n* = 7). Within the clinical trial, 34 patients were included in the control arm (i.e., doxorubicin), whereas 32 were enrolled in the experimental arm (doxorubicin plus trabectedin). Of the 66 cases included in the translational cohort, 12 (18.2%) had specific chromosomal translocations. There were 62 events of progression and 39 patients eventually died, among the patients included in the translational research. The demographic and clinicopathologic characteristics are represented in Table 1.

### 2.2. Expression of DDR-Associated Genes in STS Samples

Gene expression analysis of 4 genes (*BRCA1*, *CUL4A*, *ERCC1,* and *ERCC5*), involved in the DNA damage repair (DDR) machinery, was performed in 66 surgical tumor samples. The median absolute expression of *BRCA1*, *CUL4A*, *ERCC1,* and *ERCC5* in the whole series and both individual cohorts are shown in Table 2.

In the whole series, which included both arms of the clinical study, the expression of *CUL4A* significantly correlated with the expression of *ERCC1* (ρ = 0.668, *p* < 0.001) and *ERCC5* (ρ = 0.703, *p* < 0.001). The expression of *ERCC1* also significantly correlated with *ERCC5* expression; unexpectedly, the expression of *BRCA1* was also positively correlated with the expression of the other 3 genes—Table S1.

### 2.3. Association of *BRCA1*, *CUL4A*, *ERCC1*, and *ERCC5* with Clinical Outcome

Sixty-six patients were included in the univariate analysis with a median follow-up of 13 months—Table 3.

In the whole series, the expression of *BRCA1, CUL4A, ERCC1,* and *ERCC5* did not achieve significant correlation with PFS and OS. Similar results were observed regarding the control group, which included the patients treated with doxorubicin in monotherapy.

**Figure 1:**
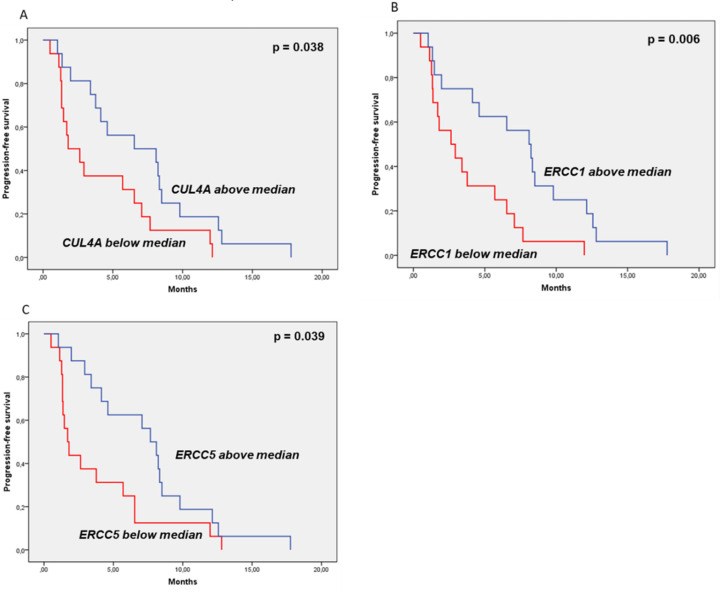
Prognostic and predictive value of DNA-damage repair genes. Samples were grouped taking into account the median of gene expression. (**A**) high expression of *CUL4A* significantly correlated with better progression-free survival (PFS) on trabectedin plus doxorubicin arm (6.53 months (95% CI: 0.00–13.39) vs 1.80 months (95% CI: 0.00–3.63); *p* = 0.038); (**B**) high expression of *ERCC1* significantly correlated with better (PFS) on trabectedin plus doxorubicin arm (8.10 months (95% CI: 4.77–11.43) vs 2.63 months (95% CI: 0.41–4.86) *p* = 0.006) and (**C**) high expression of *ERCC5* significantly correlated with better PFS on trabectedin plus doxorubicin arm (7.67 months (95% CI: 5.64–9.69) vs 1.70 months (95% CI: 1.05–2.35); *p* = 0.039).

Nonetheless, in the experimental group overexpression of *CUL4A, ERCC1,* and *ERCC5* significantly correlated with better mPFS. Amongst the transcripts analyzed, high expression of *ERCC1* was the most significantly associated with longer PFS on trabectedin plus doxorubicin arm (8.10 months (95% CI: 4.77–11.43) vs 2.63 months (95% CI: 0.41–4.86) *p* = 0.006). Likewise, high expression of *ERCC5* (7.67 months (95% CI: 5.64–9.69) vs 1.70 months (95% CI: 1.05–2.35); *p* = 0.039) and of *CUL4A* (6.53 months (95% CI: 0.00–13.39) vs 1.80 months (95% CI: 0.00–3.63); *p* = 0.038) were all associated with better PFS on the experimental group—Table 3 and Figure 1. *BRCA1* did not correlate with PFS in this arm—Table 3. Considering these translational variables as continues variables using univariate and univariate COX regression, only *ERCC1* was significantly associated to PFS (HR: 0.76; 95%CI: 0.61-0.96; *p* = 0.021).

None of these genes were statistically significant correlated with OS in the trabectedin plus doxorubicin group; however, a trend was observed in the case of *CUL4A*, where high expression showed a tendency for better OS (22.63 months (95% CI: 17.02–28.25) vs 13.53 months (95% CI 6.25–20.81); *p* = 0.059)—As observed in Figure S1.

Gene expression was also correlated with clinical variables that were reported to have prognostic value in this study [19]. In the whole series, the histologic grade negatively correlated with the expression of *CUL4A* (ρ = −0.298; *p* = 0.017), *ERCC1* (ρ = −0.321; *p* = 0.011) or *ERCC5* (ρ = −0.280; *p* = 0.025). This statistical significant negative correlation between histologic grade and *CUL4A* (ρ = −0.423; *p* = 0.018) or *ERCC1* (ρ = −0.423; *p* = 0.018) expression was maintained in the experimental group, whereas the negative correlation between histologic grade and *ERCC5* (ρ = −0.393; *p* = 0.024) was only maintained in the doxorubicin arm—Table S2.

### 2.4. CUL4A Protein Expression Analysis 

CUL4A protein expression was determined in a series of 85 patients, of which 41 cases were negative and 44 cases were positive for CUL4A immunostaining. Among the 85 cases, 44 samples were from patients treated with doxorubicin in monotherapy (*n* = 22 CUL4A positive and *n* = 22 CUL4A negative) and 41 samples derived from patients treated with the combination of trabectedin plus doxorubicin (*n* = 22 CUL4A positive and *n* = 19 CUL4A negative). An example of CUL4A immunostaining is represented in Figure S2. Demographic and clinicopathologic features of this subset of 85 cases are displayed in the Table S3.

**Figure 2:**
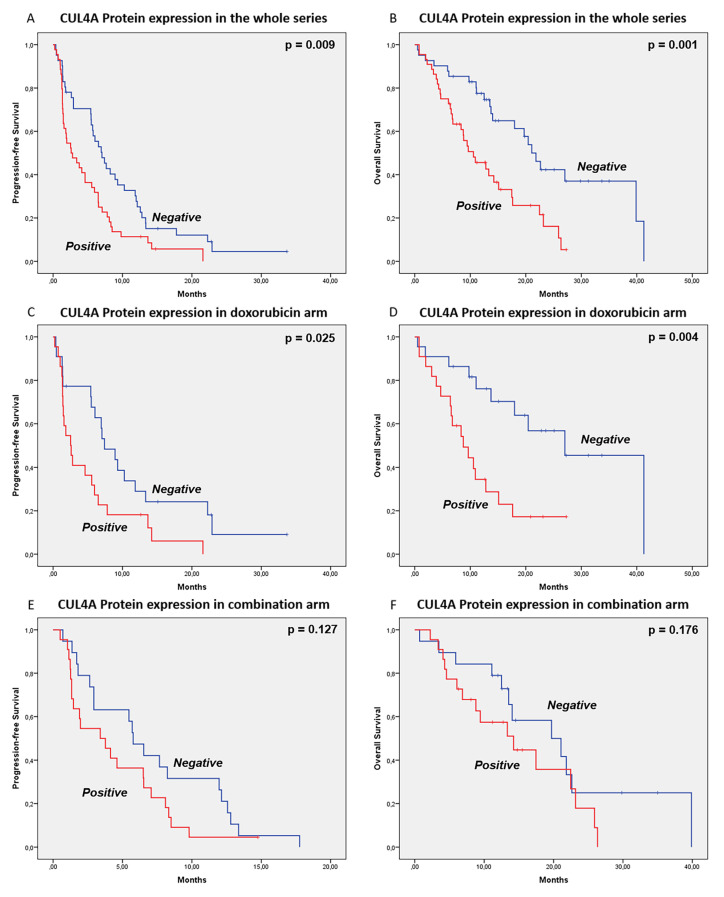
Prognostic and predictive value of CUL4A protein expression. Samples were grouped as CUL4A positive or negative, taking into account the nuclear expression levels evaluated by immunohistochemistry. Antibody: anti-CUL4A polyclonal antibody (1:50, 2699s, Cell Signaling Technology, Danvers, MA, USA). In the whole series CUL4A protein expression was associated with worse PFS (**A**): 2.60 months (95% CI: 0.58–4.62) vs 7.03 months (95% CI: 5.03–9.04), *p* = 0.009; and (**B**) and with worse OS (**B**): 10.57 months (95% CI: 5.95–15.18) vs 21.07 months (95% CI: 17.70–24.43), *p* = 0.001. In the doxorubicin arm, CUL4A expression was also associated with worse PFS (**C**): 2.53 months (95% CI: 1.12–4.00) vs 7.4 months (95% CI: 4.45–10.35), *p* = 0.025 and worse OS (**D**): 8.73 months (95% CI: 4.62–12.84) vs 27.03 months (95% CI: 16.99–37.08), *p* = 0.004. In the combination series, CUL4A protein expression did not correlate with PFS (**E**): 3.40 months (95% CI: 0.83–6.00) vs 5.77 months (95% CI: 4.25–7.28), *p* = 0.127, nor OS (**F**): 14.23 months (95% CI: 5.68–22.79) 19.70 months (95% CI: 8.82–30.58), *p* = 0.176.

Regarding the univariate analysis, positive expression of nuclear CUL4A protein was associated with worse PFS in the whole series: 2.60 months (95% CI: 0.58–4.62) vs 7.03 months (95% CI: 5.03–9.04), *p* = 0.009; and with worse PFS in doxorubicin arm: 2.53 months (95% CI: 1.12–4.00) vs 7.4 months (95% CI: 4.45–10.35), *p* = 0.025. On the other hand, CUL4A protein expression did not significantly correlate with PFS in the combination arm: 3.40 months (95% CI: 0.83–6.00) vs 5.77 months (95% CI: 4.25–7.28), *p* = 0.127—Figure 2 and Table S4.

Likewise, positive expression of CUL4A protein was significantly associated with worse OS, in the whole series: 10.57 months (95% CI: 5.95–15.18) vs 21.07 months (95% CI: 17.70–24.43), *p* = 0.001; and in doxorubicin arm: 8.73 months (95% CI: 4.62–12.84) vs 27.03 months (95% CI: 16.99–37.08), *p* = 0.004. Also, a trend was observed for worse OS in the combination arm, when CUL4A protein expression was positive: 14.23 months (95% CI: 5.68–22.79) vs 19.70 months (95% CI: 8.82–30.58), *p* = 0.176—Figure 2 and Table S4. The expression of CUL4A did not correlate with *CUL4A* gene expression in our series (Spearman’s ρ = 0.109; *p* = 0.386).

### 2.5. In Vitro Correlation between Gene Expression and Trabectedin Sensitivity

The expression of significant genes (*ERCC1*, *ERCC5,* and *CUL4A*) and its correlation with trabectedin activity were also an object of study in the pre-clinical context, with the purpose of validating the translational results.

In cell lines, the mean absolute levels of *ERCC1* were 0.0338 (0.0179–0.0685), of *ERCC5* 0.0023 (0.0006–0.0068), and of *CUL4A* 0.0126 (0.046–0.126). The expression levels of each gene, by cell line, are represented in Figure S3.

Noteworthy, high expression of *ERCC1* was significantly correlated with lower trabectedin IC_50_ values (ρ = −0.964; *p* < 0.001). Higher expression of *CUL4A* also showed a correlation trend for lower trabectedin IC_50_ values (ρ = −0.750; *p* = 0.052). The expression of *ERCC5* was not correlated with trabectedin In Vitro activity in the selected STS cell line panel (ρ = −0.143; *p* = 0.760)—Table 4.

## 3. Discussion

In this prospective study, only the expression levels of *ERCC5*, *CUL4A,* and mainly of *ERCC1* behaved as predictive biomarkers of trabectedin efficacy, supporting the relationship between DDR-associated genes and trabectedin activity.

Our data showed that high expression of *ERCC5* and mainly *ERCC1*, which are key factors in NER pathway, were related with increased anti-tumoral activity of trabectedin (i.e., PFS). Additionally, cell lines with higher levels of *ERCC1* were also more sensitive to trabectedin treatment. Meanwhile, *ERCC5* levels were not significantly correlated with trabectedin activity, in our pre-clinical studies. These results suggest that the overexpression of *ERCC1* could be a reliable positive predictive biomarker of trabectedin activity, whereas its low expression may be related with trabectedin-resistance. Indeed, resistance to trabectedin has been reported in human NER-deficient cell lines. Cells lacking functional *ERCC1* had a 2- to 8-fold increase in trabectedin IC_50_ values as compared to the parental cell line. Moreover, the ectopic expression of ERCC1 in NER-deficient cells sensitized them to trabectedin [11]. Besides, high expression levels of *ERCC1* and *ERCC5* had also been associated with improved PFS on the trabectedin line, in a retrospective series, whereas the levels of *BRCA1* were not correlated with the clinical outcome. Similar data was observed in our prospective study [8,20,21,22]. Yet, another study reported that low levels of *BRCA1* expression correlated with statistically significant better response to trabectedin [9]. It is worth mentioning that in our series patients were treated with trabectedin at the first-line of advance disease, and samples were collected at the time of diagnosis. Hence, this fact would indicate higher reliability since no systemic treatment that could induce changes in gene or protein expression, were given between diagnostic tumor biopsy time and at the baseline of the study.

The high expression of *CUL4A* was also significantly associated with better outcome in patients treated with trabectedin. These results could be related to the fact that CUL4A forms proteolytic complexes with DDBs proteins, which are relevant in the activation of NER mechanism of DNA repair [13]. Accordingly, and taking into account that trabectedin activity seems to rely at least partially on NER-efficiency [7,23,24], our results support that high expression of CUL4A should be associated with improved trabectedin activity. The potential predictive value of CUL4A has previously been reported in a panel of 10 breast cancer cell lines, where the expression of *CUL4A* was associated with higher trabectedin sensitivity [16]. Moreover, the downregulation of CUL4A in these cell lines increased resistance to trabectedin. In the same study, lower *BRCA1/ERCC5*, *BRCA1/CUL4A*, and *XRCC3/CUL4A* expression ratios were also associated with trabectedin activity; however, the ratios between *BRCA1*/*ERCC5*, *BRCA1*/*CUL4A,* or *BRCA1*/*ERCC1* were not correlated with trabectedin activity (data not shown) in our series.

Nonetheless, it is important to mention that, in our series, the results of *CUL4A* mRNA levels were not consistent with the results attained at protein level. High expression levels of CUL4A were associated with better PFS for trabectedin in nontranslocation-related sarcomas [16]; however, protein expression analysis showed an unexpected association between worse PFS and cases with high expression of CUL4A in our series. This result was significant in the whole series as well as in the doxorubicin-treated cases, while in the combination group there was also a tendency for worse PFS in samples with high protein levels of CUL4A. Of note, CUL4A has been shown to regulate the expression of *ABC* efflux pumps, more precisely multidrug resistance-associated protein 1 (MRP-1) and P-glycoprotein (P-gp) [25]. These transporters confer doxorubicin-resistance in STS, which might explain the worse PFS in doxorubicin arm associated with high CUL4A protein expression [26,27]. 

Yet, it is important to note that accurate quantification of CUL4A immunostaining was deemed to be difficult, mainly due to the lack of unique epitope and the cross-positivity with CUL4B [28]. Accordingly, CUL4A protein expression data should be carefully interpreted, since the levels of protein expression may represent both cullin E3 ligase scaffolding proteins CUL4A and CUL4B. This issue could also justify the different prognostic value of CUL4A protein and RNA.

Contrariwise, NER pathway seems to be involved in the repair of doxorubicin-induced lesions [29,30], indicating that NER-deficient tumors could be more sensitive to doxorubicin treatment. Nevertheless, our data did not show any association between the expression of NER-associated genes and the clinical outcome of patients treated with doxorubicin. These results could be justified by clinical and pre-clinical evidence, describing tissue-specific patterns of DNA repair, which in turn might be related to mutations in DDR-specific genes [31]. Hence, the heterogeneity of STS subtypes and of tumor localizations in our series, associated with other genetic factors not explored (i.e., mutational analysis) may impact the reparation of doxorubicin-induced lesions and the correlations taken from this study.

Our results also showed a statistically significant correlation between histologic grade and gene expression. High expression of *CUL4A*, *ERCC1,* and *ERCC5* correlated with low histologic grade in the wholes series and this association was also statistically significant in the experimental arm, at least for *CUL4A* and *ERCC1*. Similar to our data, high expression of *CUL4A* or *ERCC1* had been associated with better outcome in sarcomas [8,16,32,33]; however, and to our knowledge, no correlation between *CUL4A* or *ERCC1* expression levels and histologic grade had been reported in STS. Pre-clinical studies should be performed in sarcomas to address if these genes may play an anti-tumoral role in sarcomas or if they are only relevant in the mechanisms of action of doxorubicin and trabectedin. Moreover, it could be relevant to perform multivariate analysis in series with a higher number of cases and including both clinical (e.g., histologic grade and others) and translational (e.g., *CUL4A* and *ERCC1*) variables. This analysis could help validate the predictive value of these genes. In this study, only 2 variables could be considered for multivariate analysis, taking into account the number of cases included in the trabectedin plus doxorubicin arm.

Our study has however some limitations that should be taken into account. The most important is the lack of a cohort of cases treated with trabectedin in monotherapy and in which the validation of predictive biomarkers could be performed. Still and to further explore the predictive value of these molecular factors, the expression of DDR-associated genes is being currently evaluated in a separate study, using a series of 301 cases treated with trabectedin in second or further lines of advance disease [34]; with the limitation that diagnostic specimens in some cases were collected far from the time in which the patients were treated with trabectedin. Results from this study will help understand and validate the data attained in this prospective analysis. Moreover, it is important to notice that the median cut-off values used in our study to group expression data might represent a limitation. Other statistical metrics as receiver operating characteristic (ROC) curve could be considered to group continuous variables in future biomarkers studies; mostly in big series of cases, where it will be more reliable to detect a high sensitive and specific cut-off, with a meaningful clinical value. For this reason, the data obtained in this study is an interesting exploratory observation, but that should be validated in a bigger independent sample, where a more robust cut-off may arise. Another limitation is the lack of reliable antibodies for protein expression analysis in paraffin tumor samples, which limits the validation of potential biomarkers (e.g., *ERCC1* and *CUL4A*). 

## 4. Methods

### 4.1. Patients

The cases included in this translational study, for gene (*n* = 66) and protein expression (*n* = 85) analyses were collected prospectively within the randomized phase II trial of trabectedin and doxorubicin compared with doxorubicin alone as first-line treatment in patients with advanced STS All subjects gave their informed consent for inclusion before they participated in the study. The study was conducted in accordance with the Declaration of Helsinki, and the protocol was approved by the Ethics Committee of Illes Balears (EudraCT 2008-008922-55). Patients included in this trial had locally advanced non-resectable or metastatic STS, with measurable disease according to Response Evaluation Criteria in Solid Tumors (RECIST) 1.0. Additional inclusion and exclusion criteria have been previously described [19].

### 4.2. Gene Expression of Tumour Samples

One representative formalin-fixed paraffin-embedded (FFPE) block was selected from each patient and three sections of 20 μm thick were cut. For the isolation of the mRNA, RecoverAll™ Total Nucleic Acid Isolation Kit for FFPE (Ambion, Texas, TX, USA) was used according to manufacturer’s instructions. RNA concentration was measured in a NanoDrop-1000 spectrophotometer (Thermo scientific, Waltham, MA, USA). One μg of RNA obtained was used to test RNA integrity, by the presence of the 28S and 18S ribosomal bands in a 1% agarose gel electrophoresis stained with ethidium-bromide and visualized under ultraviolet light.

Reverse transcription was performed from 200 ng of total RNA using the High Capacity cDNA Reverse Transcription Kit^®^ (Applied Biosystems, Foster City, CA, USA), following manufacturers’ instructions and as described elsewhere [26].

Gene expression was measured by qRT-PCR using the following TaqMan assays on demand (Applied Biosystems, Foster City, CA, USA): *BRCA1* (Hs01556190_m1), *CUL4A* (Hs00757716_m1), *ERCC1* (Hs01012159_m1), *ERCC5* (Hs01012159_m1) in a 7500 Fast thermocycler (Applied Biosystems). Furthermore, beta-2-microglobulin (Hs99999907_m1) and GAPDH (Hs00266705_m1) were used as housekeeping genes.

Expression was then calibrated using a universal human RNA pool (Stratagene, La Jolla, CA, USA) to normalize the relative expression of the genes analyzed following the 2^−ΔΔCt^ method [35].

### 4.3. Immunohistochemistry

Two or three representative areas (1 mm in diameter) of each tumor were selected for tissue microarray (TMA) production by first examining the hematoxylin and eosin-stained tumor slide and then sampling the tissue from the corresponding paraffin blocks. A TMA instrument (Beecher Instruments; Sun Prairie, WI, USA) was used for TMA assembly. Immunohistochemistry was performed in TMAs 4-µm sections, using an anti-CUL4A polyclonal antibody (1:50, 2699s, Cell Signaling Technology, Danvers, MA, USA). Nuclear CUL4A expression was analyzed as negative and positive (positive cases were considered if they displayed staining in at least 5% of cells). CUL4A protein expression was determined in 85 tumor samples, collected at disease onset. Colorectal cancer tissue was used as positive control of CUL4A expression.

### 4.4. Cell Lines

The following STS cell lines were used for *ERCC1*, *ERCC5* and *CUL4A* gene expression analysis: Liposarcoma cell lines 93T449 (ATCC^®^ CRL-3043™; ATCC, Old Town Manassas, VA, USA) and SW872 (ATCC^®^ HTB-92™; ATCC, Manassas, VA, USA); leiomyosarcoma primary cell lines AA (kindly provided by Dr. Amancio Carnero of the Institute of Biomedicine of Seville, CSIC, US, HUVR; Seville, Spain) and CP0024 (established in Martin-Broto laboratory); SW982 (ATCC^®^ HTB-93™; ATCC) synovial sarcoma cell line; fibrosarcoma cell line HT-1080 (ATCC^®^ CCL-121™; ATCC) and uterine leiomyosarcoma cell line SK-UT-1 (ATCC^®^ HTB-114™; ATCC).

HT-1080 and AA cell line were maintained in F-10 medium (Gibco^TM^, Thermo Fischer Scientific, Waltham, MA, USA), 93T449 and CP0024 were cultured in RPMI cell medium (Gibco^TM^), SK-UT-1 was maintained in DMEM cell culture medium (Gibco^TM^) and both SW872 and SW982 were cultured in Leibovitz’s L-15 Medium (Gibco^TM^). All the cell culture mediums were supplemented with 10% FBS, and 100 units/mL penicillin (PAA) and 100 μg/mL streptomycin. Cells were checked routinely and test for contamination by Mycoplasma or fungi. All the cells lines were discarded after 2 months and new lines obtained from frozen stocks.

### 4.5. Determination of Trabectedin IC_50_ Values

Cell lines were seeded in 96-well plates and treated separately with increasing concentrations (1 × 10^−13^ M to 1 × 10^−7^ M) of trabectedin for 72 h. Cell proliferation was evaluated by MTS assay (Promega, Madison, WI, USA) and the concentrations that inhibit 50% of cell growth (IC_50_) were determined using nonlinear regression in Prism 5.0 (GraphPad Software; San Diego, CA, USA).

### 4.6. Gene Expression Determination in Cell Lines

Cells were cultured in 10 cm dishes for 48 h, time in which they were harvested for gene expression analysis. Total RNA was isolated by TRIzol^®^ (Invitrogen Corp., Carlsbad, CA, USA)—chloroform method, from all the cell lines, according to the manufacturer’s protocol. One microgram of RNA was submitted to reverse transcription using the High Capacity cDNA Reverse Transcription Kit (Applied Biosystems™—Thermo Fischer Scientific), in the presence of MultiScribe™ Reverse Transcriptase and a random primer scheme for initiating cDNA synthesis. The cDNA obtained was amplified and quantified by real-time quantitative PCR, using the GoTaq^®^ qPCR Master Mix Kit (Promega). Individual quantification of gene expression was performed using the comparative CT method (CT) and the relative expression will be calculated as 2^−ΔCT^. The following assays were used for determine gene expression: *ERCC1*; *ERCC5*; *CUL4A;* and *GAPDH*. Three biological replicates with three technical replicas each, were performed.

### 4.7. Statistical Analysis

All the categorical variables were reported as relative frequencies (%) and quantitative variables were expressed as median and ranges. OS and PFS were measured from the date of diagnosis (OS) or from the date of initial treatment within the clinical trial (PFS) to the final event, and were estimated according to the Kaplan–Meier method. The associations between the variables of interest (i.e., gene expression and clinical outcomes) were performed by the log-rank test. Univariate and multivariate COX regression was carried out with continuous translational variables. All these associations were pre-planned to be performed in the whole series and in each treatment cohort. Correlations among gene expression levels and between IC_50_ values and In Vitro gene expression were performed using Spearman’s rank correlation coefficient (ρ). All *p*-values reported were 2-sided, and the statistical significance was defined at *p* = 0.05. All the statistical procedures were performed with SPSS 22.0 software (IBM, Armonk, NY, USA).

## 5. Conclusions

High expression levels of *ERCC1*, *CUL4A,* and *ERCC5* seem to be predictive biomarkers of trabectedin activity and they were associated in our series with longer PFS for trabectedin in advanced STS. The results showed in this study support the importance of NER-efficiency on the mechanism of action of trabectedin, while it opens new roads for further research on the role of CUL4A on the activity of other chemotherapeutic agents in the context of STS. CUL4A is activated, in the DDBs complexes, by NEDD8 and this latter protein seems to be critical in the activation of NER [36]; therefore, the combination of pevonidestat, an inhibitor of NEDD8-activating enzyme (NAE) that prevents activation of cullin-RING ligases, with doxorubicin, gemcitabine or other chemotherapeutic agents that are active in NER-deficient conditions should be explored in pre-clinical experiments.

## Figures and Tables

**Table 1:** Demographics and clinical-pathologic information (*n* = 66).

Median Age (Range)	52 (21–72)
Sex:	
Female	31 (47%)
Male	35 (53%)
**Median tumor Size (mm) (Range)**	**90 (2–300)**
Histological Grade:	
1	10 (15.6%)
2	18 (28.1%)
3	36 (56.3%)
Primary tumor site	
Extremity	25 (38.4%)
Head and neck	2 (3.1%)
Trunk wall	3 (4.6%)
Retroperitoneum	15 (23.1%)
Others	20 (30.8%)
Disease type	
Localized	38 (62.3%)
Metastatic	23 (37.7%)
Sarcoma subtypes:	
Leiomyosarcoma	22 (33.3%)
Liposarcoma	12 (18.1%)
UPS *	12 (18.1%)
Fibrosarcoma	4 (6.1%)
Haemangiopericytoma	3 (4.6%)
MPNST **	3 (4.6%)
Synovial Sarcoma	3 (4.6%)
Others ***	7 (10.6%)
Experimental Arm	
Doxorubicin	34 (51.5%)
Doxorubicin plus Trabectedin	32 (48.5%)

* UPS: Undifferentiated pleomorphic sarcoma; ** MPNST: Malignant peripheral nerve sheath tumor. *** Others: Angiosarcoma (*n* = 1) and Unclassified sarcoma (*n* = 6).

**Table 2:** Gene expression results.

Gene	Median Expression ^1^ in Whole Series (Range)	Median Expression ^1^ in Control Arm (Range)	Median Expression ^1^ in Experimental Arm (Range)
*BRCA1* (*n* = 64)	0.52 (0.04–3.75)	0.47 (0.08–2.97)	0.59 (0.04–3.75)
*CUL4A* (*n* = 65)	1.31 (0.10–31.07)	1.20 (0.24–7.79)	1.46 (0.10–31.07)
*ERCC1* (*n* = 64)	1.18 (0.11–10.82)	1.14 (0.16–7.70)	1.22 (0.11–10.82)
*ERCC5* (*n* = 66)	0.37 (0.01–7.07)	0.37 (0.02–1.45)	0.39 (0.01–7.07)

^1^ 2^−ΔΔCT^, median relative expression.

**Table 3:** Survival analysis in accordance to gene expression.

Whole Series ^1^				
Biomarker	Median PFS (Months) (95% CI)	*p*	Median OS (Months) (95% CI)	*p*
*BRCA1* (*n* = 64)		0.902		0.684
Below median (*n* = 32)	4.60 (0.00–9.22)		22.47 (4.43–40.51)	
Above median (*n* = 32)	5.70 (3.02–8.38)		17.47 (12.15–22.78)	
*CUL4A* (*n* = 65)		0.173		0.343
Below median (*n* = 33)	4.60 (0.25–8.95)		14.03 (4.68–23.39)	
Above median (*n* = 32)	5.50 (2.17–8.83)		21.83 (11.62–32.05)	
*ERCC1* (*n* = 64)		0.696		0.406
Below median (*n* = 32)	3.73 (0.30–7.23)		17.47 (2.99–31.94)	
Above median (*n* = 32)	5.50 (2.87–8.13)		17.97 (10.75–25.18)	
*ERCC5* (*n* = 66)		0.559		0.593
Below median (*n* = 33)	4.60 (1.15–8.05)		17.97 (6.89–29.04)	
Above median (*n* = 33)	5.97 (1.99–9.94)		17.47 (7.38–27.56)	
**Control Group ^2^**				
**Biomarker**	**Median PFS (months)** **(95% CI)**	***p***	**Median OS (months)** **(95% CI)**	***p***
*BRCA1* (*n* = 34)		0.642		0.406
Below median (*n* = 17)	5.43 (1.18–9.69)		8.73 (-)	
Above median (*n* = 17)	6.03 (0.12–11.95)		17.97 (11.16–24.77)	
*CUL4A* (*n* = 33)		0.626		0.994
Below median (*n* = 16)	4.60 (0.00–12.70)		-	
Above median (*n* = 17)	5.50 (0.97–10.03)		15.10 (7.41–22.79)	
*ERCC1* (*n* = 32)		0.321		0.871
Below median (*n* = 16)	6.93 (3.80–10.07)		27.03 (0.00–61.26)	
Above median (*n* = 16)	2.53 (0.18–4.89)		13.73 (9.96–17.51)	
*ERCC5* (*n* = 34)		0.515		0.746
Below median (*n* = 17)	6.93 (4.78–9.09)		-	
Above median (*n* = 17)	2.60 (0.00–8.02)		13.73 (9.51–17.96)	
**Experimental Group ^3^**				
**Biomarker**	**Median PFS (months)** **(95% CI)**	***p***	**Median OS (months)** **(95% CI)**	***p***
*BRCA1* (*n* = 30)		0.420		0.608
Below median (*n* = 15)	1.70 (0.00–4.02)		14.23 (13.22–15.24)	
Above median (*n* = 15)	5.70 (0.87–10.54)		21.07 (10.37–31.77)	
*CUL4A* (*n* = 32)		0.038		0.059
Below median (*n* = 16)	1.80 (0.00–3.63)		13.53 (6.25–20.81)	
Above median (*n* = 16)	6.53 (0.00–13.39)		22.63 (17.02–28.25)	
*ERCC1* (*n* = 32)		0.006		0.295
Below median (*n* = 16)	2.63 (0.41–4.86)		14.03 (5.73–22.34)	
Above median (*n* = 16)	8.10 (4.77–11.43)		21.07 (11.32–30.81)	
*ERCC5* (*n* = 32)		0.039		0.521
Below median (*n* = 16)	1.70 (1.05–2.35)		13.53 (5.94–21.13)	
Above median (*n* = 16)	7.67 (5.64–9.69)		21.07 (15.04–27.09)	

^1^ Whole series: includes all the cases from both arms; ^2^ Control Group: Doxorubicin; ^3^ Experimental Group: Doxorubicin plus Trabectedin. The median values were calculated for each gene in the whole series and in each treatment group.

**Table 4:** Trabectedin IC50 and gene expression levels in soft-tissue sarcoma (STS) cell lines.

Cell line	IC50 (pM)	*CUL4A* *	*ERCC1* *	*ERCC5* *
93T449	156	0.0046	0.0179	0.0006
AA	107	0.0106	0.0318	0.0007
CP0024	399	0.0099	0.0252	0.0019
HT-1080	148	0.0083	0.0263	0.0022
SK-UT-1	87	0.0323	0.0685	0.0015
SW872	142	0.0124	0.0270	0.0068
SW982	90	0.0103	0.0403	0.0022
Spearman's rank correlation coefficient (ρ) **		*CUL4A* *	*ERCC1* *	*ERCC5* *
Trabectedin IC50		−0.750	−0.964	−0.143
		*p* = 0.052	*p* < 0.001	*p* = 0.760

* Absolute mean levels (∆CT); ** Spearman’s rank correlation coefficient between trabectedin IC_50_ and gene expression values.

## Supplementary Materials

The following are available online at https://www.mdpi.com/2072-6694/12/5/1128/s1, Table S1: Bivariate correlations of gene expression. (Spearman’s rank correlation coefficient (ρ)), Table S2: Correlation between gene expression and clinical variables, Table S3: Demographics and clinical-pathologic information of the subset of cases included in protein expression analysis (*n* = 85), Table S4: Univariate analysis taking into account CUL4A protein expression, Figure S1: Overall Survival taking into account the median expression of *CUL4A*, Figure S2: CUL4A protein expression, Figure S3: *ERCC1*, *ERCC5* and *CUL4A* expression in soft-tissue sarcoma cell lines.
