# Supplementary material for: CUL4A, ERCC5, and ERCC1 as Predictive Factors for Trabectedin Efficacy in Advanced Soft Tissue Sarcomas (STS): A Spanish Group for Sarcoma Research (GEIS) Study
Source: Cancers (Basel). 2020 Apr 30;12(5):1128. doi: 10.3390/cancers12051128 (PMC7281106; doi:10.3390/cancers12051128)
Supplement: Supplementary file 1 [file cancers-12-01128-s001.pdf]

cancers

MDPI

Article

CUL4A, ERCC5 and ERCC1 as predictive factors for trabectedin efficacy in advanced soft tissue sarcomas (STS). *A Spanish Group for Sarcoma Research (GEIS) Study*

David S. Moura, Paloma Sanchez-Bustos, Antonio Fernandez-Serra, María Lopez-Alvarez, José L. Mondaza-Hernandez, Elena Blanco-Alcaina, Angela Gavilan-Naranjo, Paula Martinez-Delgado, Serena Lacerenza, Paloma Santos-Fernandez, Irene Carrasco-Garcia, Samuel Hidalgo-Rios, Antonio Gutierrez, Rafael Ramos, Nadia Hindi, Miguel Taron, Jose Antonio Lopez-Guerrero and Javier Martin-Broto

Supplementary Material

Table S1. Bivariate correlations of gene expression. (Spearman's rank correlation coefficient (ϱ)).

Whole Series				
	BRCA1	*CUL4A*	*ERCC1*	*ERCC5*
BRCA1	-	$\varrho = 0.468$<br>$p < 0.001$	$\varrho = 0.689$<br>$p < 0.001$	$\varrho = 0.419$<br>$p = 0.001$
CUL4A	$\varrho = 0.468$<br>$p < 0.001$	-	$\varrho = 0.668$<br>$p < 0.001$	$\varrho = 0.703$<br>$p < 0.001$
ERCC1	$\varrho = 0.689$<br>$p < 0.001$	$\varrho = 0.668$<br>$p < 0.001$	-	$\varrho = 0.599$<br>$p < 0.001$
ERCC5	$\varrho = 0.419$<br>$p = 0.001$	$\varrho = 0.703$<br>$p < 0.001$	$\varrho = 0.599$<br>$p < 0.001$	-

Table S2. Correlation between gene expression and clinical variables.

Whole series [1]				
	BRCA1 ($n = 64$)	*CUL4A* ($n = 65$)	*ERCC1* ($n = 64$)	*ERCC5* ($n = 66$)
ECOG**	$\rho = -0.122$	$\rho = 0.023$	$\rho = -0.012$	$\rho = 0.141$
($n = 65$)	$p = 0.343$	$p = 0.859$	$p = 0.928$	p = 0.262
Grade***	$\rho = -0.191$	$\rho = -0.298$	$\rho = -0.321$	$\rho = -0.280$
($n = 64$)	$p = 0.137$	$p = 0.017$	$p = 0.011$	$p = 0.025$
L-Sarcomas****	$\rho = 0.031$	$\rho = 0.139$	$\rho = 0.094$	$\rho = 0.121$
($n = 66$)	$p = 0.806$	$p = 0.268$	$p = 0.461$	$p = 0.332$
	BRCA1 ($n = 34$)	*CUL4A* ($n = 33$)	*ERCC1* ($n = 32$)	*ERCC5* ($n = 34$)
ECOG**	$\rho = -0.189$	$\rho = -0.086$	$\rho = -0.197$	$\rho = 0.063$
($n = 34$)	$p = 0.285$	$p = 0.635$	$p = 0.279$	$p = 0.724$
Grade***	$\rho = -0.151$	$\rho = -0.125$	$\rho = -0.354$	$\rho = -0.393$
($n = 33$)	$p = 0.402$	$p = 0.495$	$p = 0.051$	$p = 0.024$
L-Sarcomas****	$\rho = -0.059$	ρ = 0.026	$\rho = 0.000$	$\rho = -0.059$
($n = 34$)	$p = 0.739$	p = 0.886	$p = 1.000$	$p = 0.739$
	BRCA1 ($n = 30$)	*CUL4A* ($n = 32$)	*ERCC1* ($n = 32$)	*ERCC5* ($n = 32$)
ECOG**	$\rho = -0.098$	$\rho = 0.044$	$\rho = 0.178$	$\rho = 0.226$
($n = 31$)	$p = 0.613$	$p = 0.816$	$p = 0.337$	$p = 0.221$
Grade***	$\rho = -0.186$	$\rho = -0.423$	$\rho = -0.423$	$\rho = -0.158$
($n = 31$)	$p = 0.333$	$p = 0.018$	$p = 0.018$	$p = 0.395$
L-Sarcomas****	$\rho = 0.205$	$\rho = 0.438$	$\rho = 0.188$	$\rho = 0.313$
($n = 32$)	$p = 0.276$	$p = 0.012$	$p = 0.303$	$p = 0.081$

[1] Whole series: includes all the cases from both arms; [2] Control Group: Doxorubicin; [3] Experimental Group: Doxorubicin plus Trabectedin. * Disease type: localized vs metastatic; **ECOG: 1 vs. 0;

***Grade 3 vs 1 and 2; ****L-Sarcomas (liposarcoma and leiomyosarcoma): Yes vs. No. Spearman's rank correlation coefficient: (ϱ).

Table S3. Demographics and clinical-pathologic information of the subset of cases included in protein expression analysis (n = 85).

Median Age (Range).	52 (19–77)
Sex:	
Female	37 (44%)
Male	48 (56%)
Median Tumor Size (mm) (Range)	91 (2–1342)
Histological Grade:	
1	13 (15.8%)
2	21 (25.6%)
3	48 (58.6%)
Primary tumor site:	
Extremity	31 (36.9%)
Head and Neck	4 (4.8%)
Trunk Wall	6 (7.1%)
Retroperitoneum	19 (22.6%)
Others	24 (28.6%)
Disease type:	
Localized	43 (55.8%)
Metastatic	34 (44.2%)
Sarcoma subtypes:	
Leiomyosarcoma	27 (31.8%)
Liposarcoma	13 (15.3%)
UPS*	14 (16.5%)
Fibrosarcoma	4 (4.7%)
Hemangiopericytoma	4 (4.7%)
MPNST**	4 (4.7%)
Synovial Sarcoma	4 (4.7%)
Others***	15 (17.6%)
Experimental Arm:	
Doxorubicin	44 (51.8%)
Doxorubicin plus Trabectedin	41 (48.2%)

* UPS: Undifferentiated pleomorphic sarcoma; **MPNST: Malignant peripheral nerve sheath tumor. *** Others: Angiosarcoma (n = 3); Fibromyxoid sarcoma (n = 1); Hemangioendothelioma (n = 1); and Unclassified sarcoma (n = 10).

Table S4. Univariate analysis taking into account CUL4A protein expression.

		WHOLE SERIES		
**Biomarker**	**Median PFS (months) (95% CI)**	**p**	**Median OS (months) (95% CI)**	**p**
CUL4A (*n* = 85)				
Negative	7.03 (5.03–9.04)	0.009	21.07 (17.70–24.43)	0.001
Positive	2.60 (0.58–4.62)		10.57 (5.95–15.18)	
		CONTROL GROUP[1]		
Biomarker	Median PFS (months) (95% CI)	p	Median OS (months) (95% CI)	p
CUL4A (n = 44)				
Negative	7.40 (4.45–0.35)	0.025	27.03 (16.99–37.08)	0.004
Positive	2.53 (1.12–4.00)		8.73 (4.62–12.84)	
		EXPERIMENTAL GROUP[2]		
Biomarker	Median PFS (months) (95% CI)	p	Median OS (months) (95% CI)	p
CUL4A (n = 41)				
Negative	5.77 (4.25–7.28)	0.127	19.70 (8.82–30.58)	0.176
Positive	3.40 (0.83–6.00)		14.23 (5.68–22.79)	

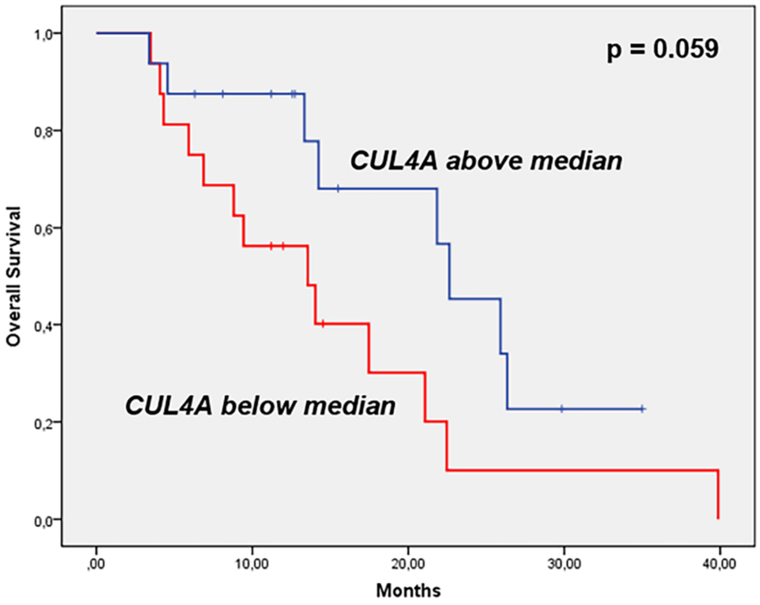

Figure S1. Overall Survival taking into account the median expression of *CUL4A*. Samples were grouped taking into account the median of gene expression. High expression of *CUL4A* showed a tendency for better OS (22.63 months (955 CI: 17.02–28.25) vs 13.53 months (95% CI 6.25–20.81); *p* = 0.059).

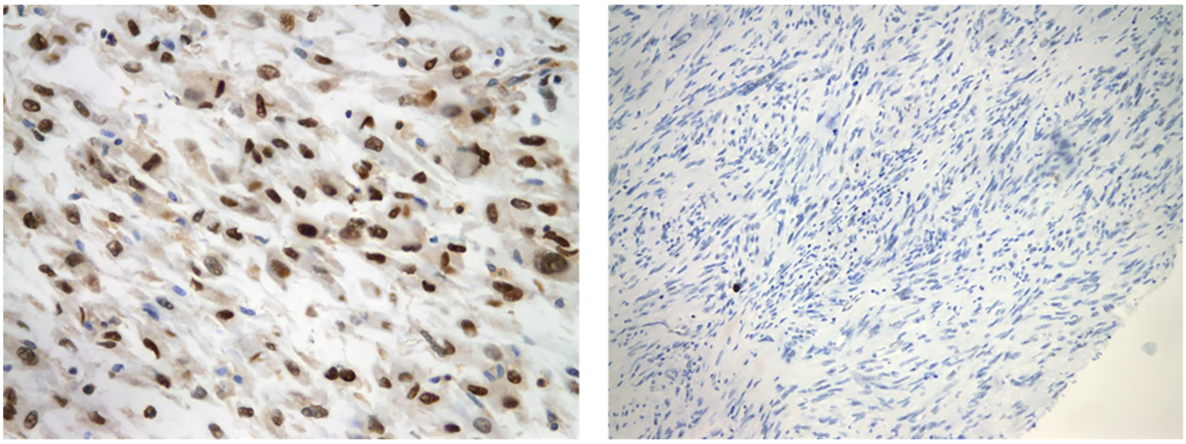

Figure S2. CUL4A protein expression. Example of CUL4A nuclear positive expression (left) and negative CUL4A expression (right). Immunohistochemistry was performed using an anti-CUL4A polyclonal antibody (1:50, 2699s, Cell Signaling Technology).

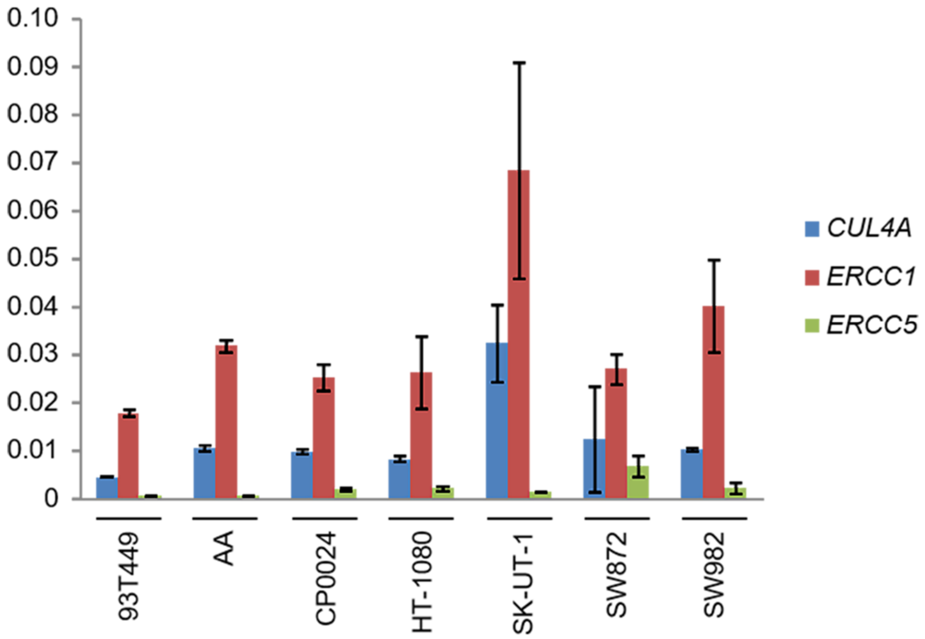

Figure S3. *ERCC1*, *ERCC5* and *CUL4A* expression in soft-tissue sarcoma cell lines. The expression levels were quantified by qRT-PCR and the expression levels relativized to the expression of *GAPDH*.
